# Lactic Acid Bacteria Isolation from Spontaneous Sourdough and Their Characterization Including Antimicrobial and Antifungal Properties Evaluation

**DOI:** 10.3390/microorganisms8010064

**Published:** 2019-12-30

**Authors:** Elena Bartkiene, Vita Lele, Modestas Ruzauskas, Konrad J. Domig, Vytaute Starkute, Paulina Zavistanaviciute, Vadims Bartkevics, Iveta Pugajeva, Dovile Klupsaite, Grazina Juodeikiene, Ruta Mickiene, João Miguel Rocha

**Affiliations:** 1 Department of Food Safety and Quality, Veterinary Academy, Lithuanian University of Health Sciences, Mickeviciaus str. 9, LT–44307 Kaunas, Lithuania; vita.lele@lsmuni.lt (V.L.); vytaute.starkute@lsmuni.lt (V.S.); paulina.zavistanaviciute@lsmuni.lt (P.Z.); ruta.mickiene@lsmuni.lt (R.M.); 2 Institute of Animal Rearing Technologies, Veterinary Academy, Lithuanian University of Health Sciences, Mickeviciaus str. 9, LT–44307 Kaunas, Lithuania; dovile.klupsaite@lsmuni.lt; 3 Microbiology and Virology Institute, Veterinary Academy, Lithuanian University of Health Sciences, Mickeviciaus str. 9, LT–44307 Kaunas, Lithuania; modestas.ruzauskas@lsmuni.lt; 4 Department of Anatomy and Physiology, Veterinary Academy, Lithuanian University of Health Sciences, Mickeviciaus str. 9, LT–44307 Kaunas, Lithuania; 5 Institute of Food Science, Department of Food Science and Technology, BOKU-University of Natural Resources and Life Sciences Vienna, Muthgasse 18, 1190 Wien, Austria; konrad.domig@boku.ac.at; 6 Department of Chemistry, University of Latvia, Jelgavas iela 1, LV-1004 Riga, Latvia; vadims.bartkevics@bior.gov.lv (V.B.); iveta.pugajeva@bior.gov.lv (I.P.); 7 Institute of Food Safety, Animal Health and Environment BIOR, Lejupesiela 3, LV-1076 Riga, Latvia; 8 Department of Food Science and Technology, Kaunas University of Technology, Radvilenu str. 19, LT-50254 Kaunas, Lithuania; grazina.juodeikiene@ktu.lt; 9 Instrumental Analysis Open Access Centre, Faculty of Natural Sciences, Vytautas Magnus University, Vileikos 8, LT-44404 Kaunas, Lithuania; 10 REQUIMTE–Rede de Química e Tecnologia, Laboratório de Química Verde (LAQV), Departamento de Química e Bioquímica, Faculdade de Ciências da Universidade do Porto (FCUP), Rua do Campo Alegre, s/n. P-4169-007 Porto, Portugal; jmfrocha@fc.up.pt

**Keywords:** spontaneous sourdough, lactic acid bacteria, *Lactobacillus*, *Leuconostoc*, *Pediococcus*, antimicrobial activity, antifungal activity, inhibition of bacterial pathogens, carbohydrate metabolism

## Abstract

This research effort aimed at isolating and phenotypically characterizing lactic acid bacteria (LAB) isolates from a spontaneous rye sourdough manufactured following traditional protocols, as well as at evaluating their antimicrobial and antifungal properties as key features for future industrial applications. Thirteen LAB strains of potential industrial interest were isolated and identified to species-level via PCR. Most of the sourdough isolates showed versatile carbohydrate metabolisms. The *Leuconostoc mesenteroides* No. 242 and *Lactobacillus brevis* No. 173 demonstrated to be gas producers; thus, revealing their heterofermenter or facultative homofermenter features. Viable counts higher than 7.0 log_10_ (CFU/mL) were observed for *Lactobacillus paracasei* No. 244, *Lactobacillus casei* No. 210, *L. brevis* No. 173, *Lactobacillus farraginis* No. 206, *Pediococcus pentosaceus* No. 183, *Lactobacillus uvarum* No. 245 and *Lactobacillus plantarum* No. 135 strains, after exposure at pH 2.5 for 2 h. Moreover, *L. plantarum* No. 122, *L. casei* No. 210, *Lactobacillus curvatus* No. 51, *L. paracasei* No. 244, and *L. coryniformins* No. 71 showed growth inhibition properties against all the tested fifteen pathogenic strains. Finally, all LAB isolates showed antifungal activities against *Aspergillus nidulans*, *Penicillium funiculosum*, and *Fusarium poae*. These results unveiled the exceptionality of spontaneous sourdough as a source of LAB with effective potential to be considered in the design of novel commercial microbial single/mixed starter cultures, intended for application in a wide range of agri-food industries, where the antimicrobial and antifungal properties are often sought and necessary. In addition, metabolites therefrom may also be considered as important functional and bioactive compounds with high potential to be employed in food and feed, as well as cosmetic and pharmaceutical applications.

## 1. Introduction

Sourdough can be defined as an acidic sharp-tasting mixture of flour (or flours) and (salted) water obtained after fermentation and used for the development of bread and other cereal-based products. These fermented doughs are very complex biological ecosystems, where lactic acid bacteria (LAB) are dominant organisms, and mostly synergistically co-existing with yeasts; the latter are well adapted to the prevailing acidic environment and able to grow to high, but mainly lower concentrations than LAB [1,2,3,4].

Many nutritional attributes of bread result considerably from the sourdough such as decreasing the risk of colorectal cancer, cardiovascular disorders, diabetes and obesity [5]. Sourdough contains a wide range of LAB, which confers positive effects on human health [6]. The metabolic activity of the sourdough microbiota strongly affects the technological performance of the dough and its nutritional properties, sensory profile, shelf-life and the overall quality of the final bread [4,7].

Several dozen species of LAB have been identified and reported in sourdoughs from all over the world, so far [4,8,9,10]. LAB are generally accepted as safe microorganisms playing important roles in food fermentation and preservation, either by the presence of natural microbiota or through the addition of starter cultures (in single cultures or as consortia of multiple microbial species) under controlled conditions [11].

The preservation effect exerted by LAB is mainly due to the production of lactic and acetic acids but also due to several other antimicrobial compounds. Sourdough fermentation results in the production of microbial metabolites that greatly contribute not only to flavor, aroma, texture, digestibility and nutritional quality of the final bread and other baking goods, but also to the food preservation. The preservation effect exerted by sourdough, and particularly by LAB, is mainly due to the production of antimicrobial (and antioxidant) metabolites, which entails important anti-bacterial and anti-fungal effects, namely organic acids (chiefly acetic and lactic acids), hydrogen peroxide, carbon hydroxide, ethanol, diacetyl, γ-aminobutyric acid, propionic acid, benzoic acid, fatty acids, bacteriocins and bacteriocin-like inhibitory substances (BLIS), among others [12].

Food/feed biopreservation is the designation for the strategies and procedures used for the preservation of food/feed using selected non-pathogenic safe microorganisms (i.e., protective microbial cultures). Such protective microorganisms are employed to prevent the development of undesirable microorganisms (through growth inhibition or killer effects); thus, protecting food/feed from mold and bacterial spoilage, increasing shelf-life and reducing food/feed losses, and improving substantially the food/feed safety. Such strategies are considered natural and effective means to control food/feed-borne pathogens [13]. Among the biopreservation strategies, LAB are considered good candidates because they produce natural antimicrobial and antioxidant metabolites. Besides, they are present in several food products as desirable natural microbiota and are recognized as non-hazardous to human health, mainly are classified as Generally Recognized As Safe (GRAS) in the USA and several LAB species fulfill the criteria of Qualified Presumption of Safety (QPS) in Europe [14,15]. It is well established that LAB of different origins can possess strong antimicrobial properties, and that they can be used in combination with antimicrobial compounds from berries and fruits as well as essential oils to increase antimicrobial activity [16,17,18,19]. Also LAB isolated from sourdough can be employed as a starter cultures towards to improve the safety of food [20,21,22,23], to provide added value [18,19,20], to increase feed functionality [24,25,26], and to design and apply antifungal coatings and films for food and feed applications [23,27].

The aim of this study was to isolate and to characterize LAB strains from spontaneous fermented rye sourdough, and to evaluate their antimicrobial and antifungal properties.

## 2. Materials and Methods

### 2.1. Materials Used for the Preparation of Sourdough

Rye flour (type 1370, falling number ˃130 s, and ash 1.31%) was obtained from Kauno Grudai Ltd. mill (Kaunas, Lithuania). Acetic acid (1%, *w/v*) and sodium chloride (1%, *w/v*) were purchased from Sigma-Aldrich (Taufkirchen, Germany).

### 2.2. Spontaneous Rye Sourdough Preparation and Sampling

Spontaneous rye sourdough was prepared by using the following procedure. Rye flour (100 g) was mixed with 150 mL of tap water (at room temperature), 1 mL acetic acid (1%, *v/v*), and NaCl (1%, *v/v*) was added till pH 4.0, and kneaded by hand during 5 min–thus giving rise to a dough yield (DY) of 250, i.e., the dough to flour weight ratio × 100 (which correlates with water activity. Resulting dough was allowed to stand for fermentation during 48 h at 30 °C in a thermostat (Memmert GmbH, Schwabach, Germany) followed by the addition of 50 g rye flour and 50 mL tap water (DY = 233.3). Finally, the fermentation was continued for an additional of 24 h at 30 °C. Aliquots of the resulting sourdough were used for in the microbiological analysis towards LAB isolation.

### 2.3. Culture Media and Microbiological Growth and Enumeration

For the enumeration and isolation of LAB de Man, Rogosa and Sharp agar (MRS) was purchased from Biolife (Milan, Italy). The culture medium was supplemented with cycloheximide (Sigma-Aldrich (Taufkirchen, Germany) to prevent growth of yeasts and molds. The pH of the culture media was adjusted to the desired value of 6.4 ± 0.2 at 25 °C. The culture media was further autoclaved after previous dissolution, under stirring, to boiling point 121 °C. The media was cooled to 50 °C and the non-thermostable cycloheximide was aseptically added to the culture media through a 0.22-µm membrane filter (Whatman, Maidstone, UK) and stirred. Duplicates of 10 g-samples of sourdough were suspended in 90 mL of sterile 2% (*w/v*) NaCl solution (Sigma-Aldrich (Taufkirchen, Germany), aseptically homogenized in a homogenizer (Bosch GmbH, Stuttgart, Germany) till pure consistency. The results were expressed as log of colony-forming units (CFU) per gram of sample. The logarithmic transformation was necessary for stabilization of variance and normalization of residuals. Analysis was performed according to method described by Bartkiene et al. [25].

### 2.4. Isolation, Atype Identification of Sourdough Lactic Acid Bacteria Strains

The 16S rDNA sequencing was conducted by applying the primer set [Bak4 (5’-AGG AGG TGA TCC ARC CGC A-3’); Bak11 (5’-AGT ATTG ATC MTG GCT CAG-3’)] and the PCR protocol as described by Di Cello et al. [28]. The RAPD PCR products were separated with agarose (2%, *w/v*) electrophoresis. The molecular fingerprinting of the all final strains was also done by rep-typing with the primer (GTG)_5_ (5′-GTG GTG GTG GTG GTG-3′). The PCR products were purified applying the peqGold Cycle-Pure Kit (Peqlab Biotechnology GmbH, Erlangen, Germany) and sequenced (Eurofins MWG Operon, Ebersberg, Germany). The received sequences were analyzed with the BLASTn tool (http://blast.ncbi.nlm.nih.gov), and a minimum sequence identity of 98% was chosen as the criterion for species identification. The PCR-based identification of the genus and species was performed according to the references given in Table 1.

### 2.5. Phenotype Characterization of the Isolated Sourdough Lactic Acid Bacteria Strains

The metabolism of several carbohydrates by sourdough LAB strains was determined by using API 50 CHL galleries (BioMérieux, Marcy-l’Etoile, France) according to the manufacturer instructions. Gas production was detected by Durham tube method [37] in MRS broth (Biolife, Milan, Italy) for 24 h at 30 °C. The growth performance of strains was monitored at 10, 30, 37 and 45 °C for 24 h in an MRS broth using a Thermo Bioscreen C automatic turbidometer (Labsystems, Helsinki, Finland). The viability of the isolated strains to grow in acidic environments was evaluated in MRS broth acidified to a final pH of 2.5 with HCl (Biolife, Milan, Italy) in tubes, according to Lee et al. [38]. Total viable counts were determined by using standard plate count techniques [39]. The results were expressed as log of colony-forming units (CFU) per milliliter. All phenotype analyses were carried out in triplicate.

### 2.6. Antimicrobial Activity Testing of the Lactic Acid Bacteria Strains by Agar Well Diffusion Technique and Liquid Medium Based Methodology

All the 13 LAB strains, *Leuconostoc mesenteroides* No. 225, *Lactobacillus plantarum* No. 122, *Enteroccocus pseudoavium* No. 242, *Lactobacillus casei* No. 210, *Lactobacillus curvatus* No. 51, *Lactobacillus farraginis* No. 206, *Pediococcus pentosaceus* No. 183, *Pediococcus acidilactici* No. 29, *Lactobacillus paracasei* No. 244, *Lactobacillus plantarum* No. 135, *Lactobacillus coryniformis* No. 71, *Lactobacillus brevis* No. 173, and *Lactobacillus uvarum* No. 245, were assessed for their antimicrobial activities against a variety of pathogenic and opportunistic bacterial strains. *Klebsiella pneumoniae*, *Salmonella enterica* 24 SPn06, *Pseudomonas aeruginosa* 17-331, *Acinetobacter baumanni* 17-380, *Proteus mirabilis*, methicillin-resistant *Staphylococcus aureus* (MRSA) M87fox, *Enterococcus faecalis* 86, *Enterococcus faecium* 103, *Bacillus cereus* 18 01, *Streptococcus mutans*, *Enterobacter cloacae*, *Citrobacter freundii*, *Staphylococcus epidermidis*, *Staphylococcus haemolyticus*, *Pasteurella multocida*, by using the agar well diffusion and minimum inhibitory concentration (MIC) methods [40,41].

The tested LAB strains were inoculated in MRS broth (Biolife, Milan, Italy) and incubated at 30 °C for 24 h. After incubation, 2 mL of the MRS broth (*v/v*), in which the LAB strains were multiplied, were inoculated into fresh MRS broth (Biolife, Milan, Italy) and propagated at 30 °C for 18 h. Afterwards, the multiplied LAB were used for the determination of their antimicrobial activities against the pathogenic and opportunistic bacterial strains listed above.

The agar well diffusion assay was used for the antimicrobial activity testing of the LAB supernatants (supernatant sample was adjusted to pH 6.5 with 1 M NaOH to eliminate the organic acid). Analysis was performed according to method described by Bartkiene et al. [16]. In addition, ability of LAB to inhibit pathogens in liquid medium was evaluated. With this aim, 0.1 mL of pathogens (previously suspended in physiological solution up to 0.5 McFarland Units) were transferred in tubes with 4.4 mL Mueller Hinton Broth (Oxoid, Basingstoke, UK) and afterwards 0.5 mL of LAB suspension (8.56 log_10_ CFU/mL) was added. The same procedure was performed using 1.0 mL of LAB with the aim to test two different LAB concentrations. Tubes were incubated for 48 h at +35 °C. The results were evaluated according to the presence/absence of visible growth. Experiments were performed in triplicate.

### 2.7. Evaluation of Antifungal Activity of the Isolated Sourdough Lactic Acid Bacteria

The antifungal activities of the LAB were determined against seven different species, viz. *Aspergillus fischeri*, *Aspergillus nidulans*, *Penicillium oxalicum*, *Penicillium funiculosum*, *Fusarium poae*, *Alternaria alternata* and *Fusarium graminearum*. These molds were previously isolated from grain-based food and were provided by the collection of the Lithuanian University of Health Sciences (Kaunas, Lithuania). All these microorganisms were cultivated on yeast extract, peptone and dextrose (YEPD) agar medium at 25 °C, in the thermostat for 5 days. The antifungal activity of LAB strains was tested by the agar well diffusion assay [41].

### 2.8. Statistical Analysis

The results were expressed as the mean (*n* = 3) ± standard deviation. Non-parametric Kruskal Wallis test followed by Dunn’s post hoc tests were used for data analysis. *p* ≤ 0.05 was considered statistically significant. Statistics were performed with SPSS for Windows XP V15.0 (SPSS, Inc., Chicago, IL, USA, 2007).

## 3. Results and Discussion

### 3.1. Genotype Identification of Lactic Acid Bacteria Strains Isolated from Spontaneous Rye Sourdough

Bands of the isolated sourdough LAB genus are shown in Table 2. Thirteen LAB strains were identified, chiefly *Leuconostoc mesenteroides, Lactobacillus plantarum, Enteroccocus pseudoavium, Lactobacillus casei, Lactobacillus curvatus, Lactobacillus farraginis, Pediococcus pentosaceus, Pediococcus acidilactici, Lactobacillus paracasei, Lactobacillus plantarum, Lactobacillus coryniformis, Lactobacillus brevis*, and *Lactobacillus uvarum*. The LAB community found in the sourdough after a spontaneous fermentation is mainly brought about by the adventitious microbiota existing in the flour or flours [1,2,3,4,10]. Sourdough microbial composition can be more or less stable for years [42].

Metabolic activity and microbial stability are key factors to ensure reproducibility between batches; therefore, to ensure the quality of sourdough bread and/or any other fermentation process that resorts to mother-dough (or sour ferment) or to microorganisms isolated therefrom as microbial starter culture. However, the microbial profile and microbial dynamic found in the sourdough depends largely on the ecological/environment conditions prevailing throughout time, from the preparation of the baking dough until the end of the fermentation process. Spontaneous sourdough fermentation and the employed environmental conditions plays a major effect upon the various microbial groups and species prevailing at the end of such a fermentative process. In a developed sourdough, only some species well-adapted to the rigid environmental conditions, prevailing during fermentation (i.e., low temperatures, high relative humidity, high total titratable acidity; presence of different antimicrobial and antioxidant metabolites, etc), became dominant. In fact, the competitive and synergetic consortia of acid tolerant yeasts and LAB usually reach rapidly viable counts above those of the adventitious microbiota initially present in the flour or flours. Nevertheless, other ubiquitous microorganisms present in the flours are likely to stay viable in some sourdoughs [1,2,3,4,10]. Previous researchers have shown that microbial diversity in sourdoughs can varied according to different geographic location [43]. It was published that *Weissella cibaria* and *Lactobacillus sanfranciscensis* were predominant in the microbiota of jiaozi and type I sourdoughs, respectively [44]. In Japan different species of LAB such as *Lactobacillus brevis, Lactobacillus alimentarius, Lactobacillus pentosus, Lactobacillus vaccinostercus, Lactobacillus sanfranciscensis,* and *Lactobacillus sakei* were detected, as well as the yeasts primarily included *Saccharomyces cerevisiae*, with *Candida humilis* in some samples [45]. Also, Chinese traditional sourdoughs from different regions were studied, and the results showed that the West group was significantly different from the North and South groups in the dominant genera (mainly *Lactobacillus*, *Pediococcus*, and *Leuconostoc*) [46]. About the LAB diversity of wheat sourdoughs collected in Ya’an city was published, from which two hundred nineteen LAB strains were isolated, and genotypic characterization indicated that the isolated LAB strains included *Lactobacillus plantarum*, *L. pantheris*, *Leuconostoc citreum*, *Weissella viridescens*, *Leu. pseudomesenteroides*, *Lactococcus lactis*, *L. raffinolactis*, and *Leu. mesenteroides* [47]. Study about the microbial diversity of the traditional Chinese sourdough showed that the predominant microbes in sourdough were *Lactobacillus*, *Pediococcus*, and *Wickerhamomyces* [48]. In spontaneous fermented chia sourdough, besides among identified LAB by culture-dependent approach, species from genus *Enterococcus* were the most abundant, as well as *Lactococcus* (*Lc. lactis*), *Lactobacillus* (*L. rhamnosus*), and *Weissella* (*W. cibaria*) species were also isolated [49]. In sourdough, as well as maize and rye flours from several geographic locations in Portugal predominant yeasts were *Saccharomyces cerevisiae* and *Candida pelliculosa*, as well as the most frequently isolated LAB were (heterofermentative) *Leuconostoc* spp. and (homofermentative) *Lactobacillus* spp.; *L. brevis*, *L. curvatus*, and *L. lactis* ssp. *lactis* for the *Lactobacillus genera*; *Lactococcus lactis* ssp. *lactis* for *lactococci*; *Enterococcus casseliflavus*, *E. durans*, and *E. faecium* for *enterococci*; and *Streptococcus constellantus* and *S. equinus* for *streptococci* [10]. Finally, sourdough has a complex microbiota that is affected by multiple factors including factors related to cereal plants, grains, and sourdough processing techniques [9].

Furthermore, during prolonged fermentation processes the variety of LAB (and other) strains tends to the greatly reduced: from several LAB species initially contained in a dough, only a few become dominant and viable at the end. Furthermore, LAB species, which do not remain viable in sourdough during the high temperatures employed during the baking processes, exhibit unique desirable technological, antimicrobial, antifungal, probiotic, biodegradation, absorption and adsorption properties, among others. Based on the above description, the isolation of the sourdough LAB in the first stages of fermentation (where higher microbial diversity is found) can prove to be very promising for the industries with special needs for microorganism with such antimicrobial, antifungal and other abilities. For this reason, all the isolated thirteen LAB strains were used for the further analysis, so as a maximum diversity of strains could be guaranteed.

### 3.2. Carbohydrate Metabolism, Gas Production, and Viability and Growth Performance at Different Temperatures and Low pH Values of Sourdough Isolates

The carbohydrate metabolism, gas production, tolerance to temperature and low pH conditions of the LAB isolated from sourdough are shown in Table 3. The carbohydrate metabolism was studied for 47 different carbon sources (Table 3). The profile of the carbohydrate fermentation capacity varied according to the sourdough LAB strain, and in the following decreasing order of No. of fermented carbohydrates: *L. paracasei* No. 244 showed activity to ferment 28 out of 47 carbohydrates, *L. plantarum* No. 122 and No. 135 strains–27; *L. casei* No. 210–24; *L. coryniformins* No. 71, *L. uvarum* No. 245, and *L. curvatus* No. 51–23; *Leu. mesenteroides* No. 242 and *P. pentosaceus* No. 183–21; *Leu. mesenteroides* No. 225–17; *L. faraginis* No. 206–10; *L. brevis* No. 173–8.

Conversion of carbohydrates into lactic and acetic acids by LAB is one of the most important fermentation processes employed in cereal-based products technologies. With the advent of pure (commercial) starter cultures for fermentation processes, it became possible to control more effectively the microbial metabolic activities and the fermentation process as a whole and, consequently, to improve food quality and safety, as well as to enhance the extending of its use to novel and a large number of biotechnological processes. The experimental information on LAB carbohydrate metabolism, as depicted in Table 3, is of utmost importance to accurately evaluate and modelling the dynamics of single/co-culture fermentations and to optimize their environmental and growth conditions to improve technological, nutritional and health attributes [16,50].

According to the carbohydrate metabolic pathways, LAB can be classified into homofermentative, facultative homofermentative or obligate heterofermentative. Depending on the type of species and strains belonging to the LAB group, they can metabolize carbohydrates into different metabolites, chiefly: *Pediococcus* and *Lactobacillus* (homofermentative) to DL or L(+) lactic acid (depending on the species, *Lactobacillus* can be homofermenters or facultative or obligate heterofermenters), *Leuconostoc* (obligate heterofermentative) to CO_2_, acetate, and D(−) lactic acid. However, it should be emphasized that the metabolic pathways for carbohydrates can be changed by the same microbial strain throughout time, depending on the environment and growth conditions, for instance during the depletion of certain substrates and the production of metabolites that became substrates to the others throughout a batch or fed-batch fermentation–thus making difficult the full elucidation and control of metabolic pathways. As examples, the metabolic pathways will depend in the complexity of the growth medium, temperature, time, water activity, and acidity, interactions between microorganisms in co-culture starters or natural sourdough systems, among many other variables [1,2,3,4,10]. Yet and particularly, when complex substrates (containing other substrates rather than the fermentable hexoses) are present in a growth medium, its fermentation may yield organic acids, acetate and CO_2_ at distinct ratios–which is very often to occur in industrial processes. Finally, most of the sourdough LAB strains in the present investigation exhibited versatile carbohydrate metabolisms–thus envisaging high potential attributes for their application in industry, for example in many fermentation processes or to be employed in recovery technologies intended for the valorization of by-products, residues and agri-food wastes resulting from the agriculture or industry.

According to the results (Table 3), from the 13 isolated sourdough LAB strains, gas production was detected in only two, *viz*. *Leu. mesenteroides* No. 242 and *L. brevis* (Group III lactobacilli). Such findings are in agreement with the expected results since both species are known to be obligate heterofermenters.

Analysis of the growth performance of the isolated sourdough LAB at different temperatures (Table 3) revealed that only the *L. casei* No. 210 strain was able to display weak growth (+) at the lowest temperature (10 °C). The activity at low temperatures is an important characteristic, since the microbial growth may be desirable or, inversely, undesirable depending on the application on demand. As example, during the storage of semi-fluid (type I and II sourdoughs) or other non-freeze-dried starter cultures, low or absence of growth activities are desirable. Conversely, it becomes undesirable when the intent is the bioconversion of materials or compounds in processes undertaken in low temperature regimes.

Furthermore, the highest growth rate at 30 °C was observed for *L. coryniformins* No. 71 and *L. casei* No. 210 strains (+++). Regarding the growth at 37 °C, the highest yields were observed for *L. casei* No. 210 (+++). Lastly, respecting to the growth highest temperature under scrutiny (45 °C), it was observed moderate growth for *L. farraginis* No. 206 (++).

With respect to the ability of selected sourdough LAB to grow under acidic environments, the highest concentration of viable cells after 2 h incubation at pH 2.5 was found to *L. paracasei* No. 244 (9.29 ± 0.1 log_10_ CFU/mL). Good microbial viability at low pH was observed for *L. casei* No. 210, *L. brevis* No. 173, and *L. farraginis* No. 206 strains–for which the concentration was higher than 8.0 log_10_ (CFU/mL), whereas viable counts higher than 7.0 log_10_ (CFU/mL) was found for *P. pentosaceus* No. 183, *L. uvarum* No. 245, and *L. plantarum* No. 135 strains after 2 h incubation at pH 2.5. Other isolated LAB strains showed significantly lower tolerance to the same acidic conditions, with values lower than 6.0 log_10_ (CFU/mL), or even absence of growth in the case of *L. coryniformins* No. 71. It is generally accepted that an isolate with full tolerance to pH 3.0 for 3 h can be considered as high-acid-resistant strain with promising probiotic properties [51,52].

In the event of microorganisms belonging to the LAB group, acid stress is a self-imposed condition, once lactic acid is the major end-product of carbohydrate metabolism and plays a major role in their competitiveness as antimicrobial agents against other microorganisms [36].

Low pH values damage both the cell wall and cell membrane; thus, influencing the membrane potential, which leads to undesirable metabolic processes, energy depletion and, eventually, to cell death [4]. Adaptation of the LAB to low pH conditions depends on their phenotype characteristics and the environmental conditions, in which cells are transiently exposed to mild nonlethal stress conditions, which, in turn, drive to an increased survival ability after a subsequent lethal challenge to the same stress [53]. The molecular mechanisms underlying transient adaptation and habituation to a specific stress may overlap to a certain degree, but they are not completely identical [53,54,55]. This may explain a number of contradictory results reported for some LAB species [56,57].

### 3.3. Antimicrobial Activity of the Isolated Lactic Acid Bacteria Strains

**Figure 1:**
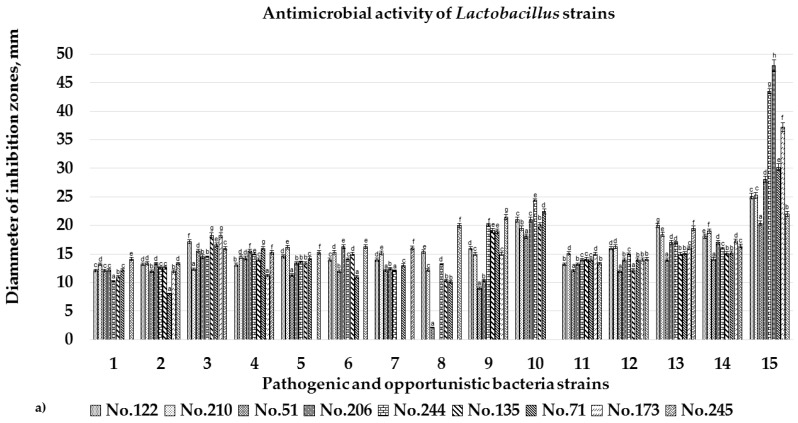

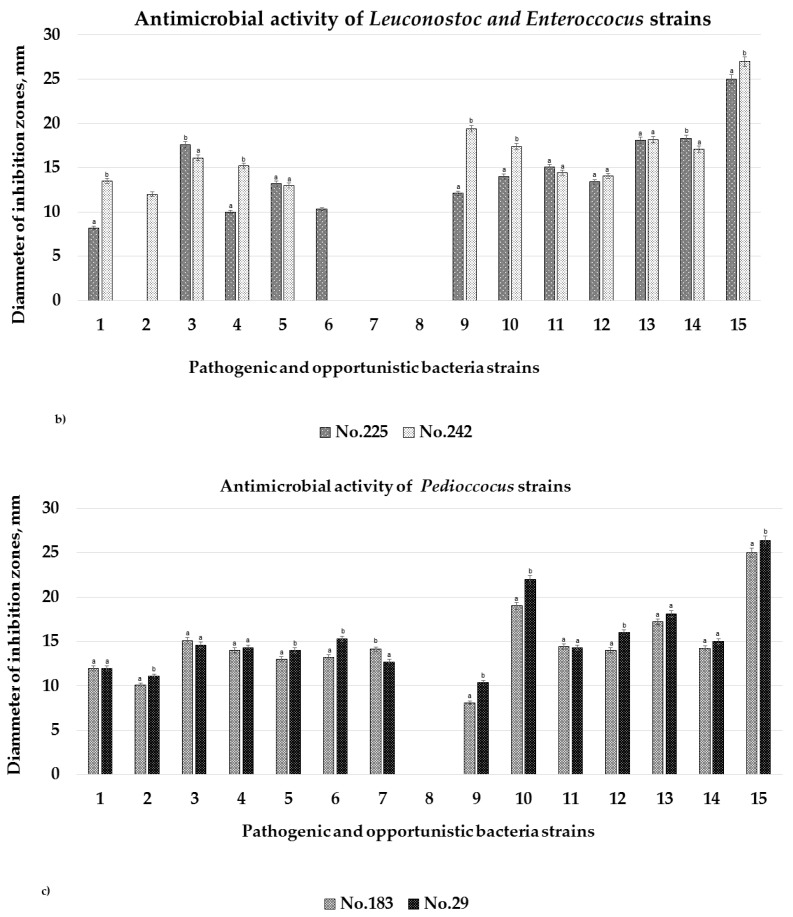
(**a**–**c**). Diameter inhibition zones (DIZ, mm) (Y axis) of the isolated sourdough lactic acid bacteria (LAB) strains against pathogenic and opportunistic microorganisms. Data expressed as mean values (*n* = 3) ± standard deviation (STDV). The isolated sourdough LAB encompasses: *Leuconostoc mesenteroides* No. 225; *Lactobacillus plantarum* No. 122; *Enteroccocus pseudoavium* No. 242; *Lactobacillus casei* No. 210; *Lactobacillus curvatus* No. 51; *Lactobacillus farraginis* No. 206; *Pediococcus pentosaceus* No. 183; *Pediococcus acidilactici* No. 29; *Lactobacillus paracasei* No. 244; *Lactobacillus plantarum* No. 135; *Lactobacillus coryniformis* No. 71; *Lactobacillus brevis* No. 173; *Lactobacillus uvarum* No. 245. The pathogenic/opportunistic bacteria (X axis) under scrutiny were: **1**–*Klebsiella pneumoniae*; **2**–*Salmonella enterica* 24 SPn06; **3**–*Pseudomonas aeruginosa* 17-331; **4**–*Acinetobacter baumanni* 17-380; **5**–*Proteus mirabilis*; **6**–MRSA M87fox - MRSA–Methicillin-resistant; **7**–*Enterococcus faecalis* 86; **8**–*Enterococcus faecium* 103, **9**–*Bacillus cereus* 18 01; **10**–*Streptococcus mutans*; **11**–*Enterobackter cloacae*; **12**–*Citrobacter freundii*; **13**–*Staphylococcus epidermidis*; **14**–*Staphylococcus haemolyticus*; **15**–*Pastaurella multocida*. ^a–h^ Mean values with different letters are significantly different (*p* ≤0.05).

Diameter inhibition zones (DIZ) of the sourdough LAB strains against pathogenic and opportunistic microorganisms are shown in Figure 1a–c. We observed inhibition properties against all the tested fifteen pathogenic and opportunistic bacterial strains by *L. plantarum* No. 122, *L. casei* No. 210, *Lactobacillus curvatus* No. 51, *L. paracasei* No. 244, and *L. coryniformins* No. 71–and from which the highest DIZ was attained against *Pasteurella multocida* (DIZ of 28.9 mm on average against *Pasteurella multocida*).

Sourdough LAB strains *L. farraginis* No. 206, *P. pentosaceus* No. 183, *P. acidilactici* No. 29, *L. plantarum* No. 135, and *L. uvarum* No. 245 displayed inhibition properties against 14 pathogenic/opportunistic bacterial strains. On the other hand, *L. farraginis* No. 206, *P. pentosaceus* No. 183 and *P. acidilactici* No. 29 did not show inhibition properties against *Enteroccoccus faecium*, whereas *L. plantarum* No. 135 could not inhibit *Enteroccoccus faecalis*, and *L. uvarum* No. 245 showed no efficiency in inhibiting *Streptococcus mutans*.

Sourdough LAB strains *Leu. mesenteroides* No. 225 and *Ent. pseudoavium* No. 242, exhibited inhibition properties against 12 of the 15 tested pathogenic/opportunistic bacterial strains. Nevertheless, lack of inhibitory ability was detected for *Leu. mesenteroides* No. 225 against *Salmonella enterica*, *Enteroccoccus faecalis*, and *Enteroccoccus faecium*, as well as for *Ent. pseudoavium* No. 242 against methicillin-resistant *Staphylococcus aureus*, *Enteroccoccus faecalis*, and *Enteroccoccus faecium*.

Among isolated sourdough LAB, *L. brevis* No. 173 strain exhibited the weakest antimicrobial properties, as this strain showed inhibition properties against 9 of the 15 tested pathogenic/opportunistic bacterial strains. Nonetheless, when comparing all the 13 isolates of sourdough LAB, *L. brevis* No. 173 strain provided the strongest antimicrobial activities (i.e., the highest DIZ values) against *Klebsiella pneumonia* (DIZ 14.1 ± 0.2 mm), *Proteus mirabilis* (DIZ 15.3 ± 0.2 mm), *Enteroccoccus faecalis* (DIZ 16.1 ± 0.3 mm), *Enteroccoccus faecium* (DIZ 20.0 ± 0.5 mm), *Bacillus cereus* (DIZ 21.5 ± 0.3 mm), and *Streptococcus epidermidis* (DIZ 19.5 ± 0.4 mm).

**Figure 2:**
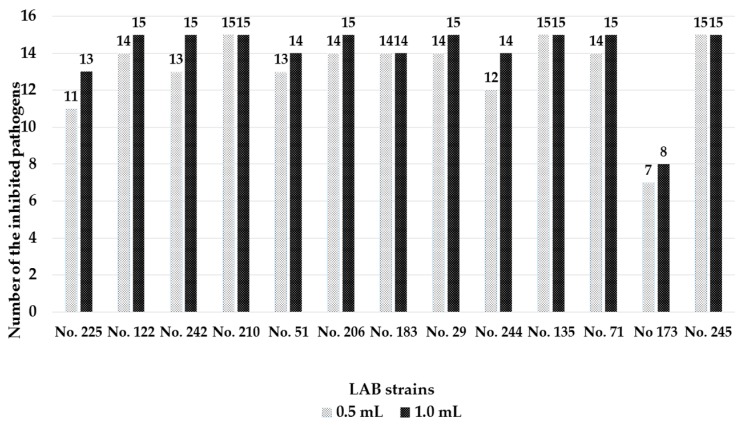
Antimicrobial activities of the tested lactic acid bacteria (LAB) strains and number of inhibited pathogenic opportunistic microorganisms in liquid medium. The isolated sourdough LAB encompasses: *Leuconostoc mesenteroides* No. 225; *Lactobacillus plantarum* No. 122; *Enteroccocus pseudoavium* No. 242; *Lactobacillus casei* No. 210; *Lactobacillus curvatus* No. 51; *Lactobacillus farraginis* No. 206; *Pediococcus pentosaceus* No. 183; *Pediococcus acidilactici* No. 29; *Lactobacillus paracasei* No. 244; *Lactobacillus plantarum* No. 135; *Lactobacillus coryniformis* No. 71; *Lactobacillus brevis* No. 173; *Lactobacillus uvarum* No. 245.

Antimicrobial activities of sourdough LAB strains at 2 different levels of concentration–0.5 mL sourdough LAB (8.56 log_10_ (CFU/mL) or 1.0 mL sourdough LAB (8.56 log_10_ (CFU/mL) and 0.1 mL (0.5 McFarlands Unit) pathogen–against pathogenic/opportunistic microorganisms in liquid culture medium are shown in Figure 2. At the lowest level of sourdough LAB (i.e., 0.5 mL sourdough LAB + 0.1 mL pathogen), the strains with the ability to inhibit the highest number of pathogens were found to be *L. casei* No. 210, *L. plantarum* No. 135, and *L. uvarum* No. 245 strains (inhibited all the tested pathogens). Furthermore, strains of *L. farraginis* No. 206, *P. pentosaceus* No. 183, *P. acidilactici* No. 29, and *L. coryniformins* No. 71 inhibited 14 of the 15 analyzed bacterial pathogens (*L. farraginis* No. 206 and *P. pentosaceus* No. 183, *P. acidilactici* No. 29, and *L. coryniformins* No. 71 only could not inhibit MRSA, *Proteus mirabilis*, *Klebsiella pneuminiae*, respectively). Moreover, the strains *Ent. pseudoavium* No. 242 and *L. curvatus* No. 51 inhibited 13 of the 15 analyzed pathogenic strains, whereas the lowest antimicrobial activity was observed for *L. brevis* No. 173–which inhibited 7 of the 15 analyzed pathogenic bacterial strains. When increasing the level of sourdough LAB inoculum (i.e., 1.0 mL sourdough LAB + 0.1 mL pathogen), most of the strains unfolded the capacity to inhibit a broader spectrum of pathogens (Figure 2)**.** However, increased concentration of *P. pentosaceus* No. 183 proved to be still not effective enough to inhibit MRSA.

The selection of appropriate LAB strains intended for in situ production of antimicrobial compounds by fermentation and the application of the purified states of such metabolites as biological food preservatives in sectors as large as food and feeds, nutraceuticals (dietary supplements and food additives), cosmetics and pharmaceuticals, is very promising.

The preservative effect of fermented products is mainly due to the acidic conditions, which are formed during LAB conversion of carbohydrates into organic acids; chiefly, lactic and acetic acids. The acidification is not exclusive to LAB, but other microorganisms may also be mostly involved in the acidification of fermentation food products, such as the group of acetic acid bacteria producers (from the *Acetobacter* species). Yet, the inhibitory action of LAB is not limited to lactic and acetic acids and a wide range of other metabolites excreted to the growth medium may possess antagonistic properties against several prokaryotic bacteria and/or eukaryotic yeasts and molds. As already cited, among those metabolites are formic acid, free fatty acids, ammonia, ethanol, hydrogen peroxide, diacetyl, acetoin, 2,3-butanediol, acetaldehyde, benzoate, bacteriolytic enzymes, bacteriocins and BLIS, as well as several other less known inhibitory substances [1,3,4,10,41,58,59,60,61,62,63,64]. Furthermore, the antimicrobial effect of LAB can also be significantly influenced by numerous physical, chemical and nutritional environmental factors [65,66].

Under this context, sourdough LAB isolated in this research work could be used to design specific starter cultures or to produce antimicrobial metabolites (among many other commercially high-added value compounds). Actually, the applications of LAB individually or as co-cultures in the prevention of bacterial and mold food spoilage represent major challenges to the industry. As a matter of fact, the production of industrial single/mixed starter cultures still needs further and deep investigations to address the incipient existing knowledge concerning the metabolic activity and microbial dynamic in such complex biological systems, but also to find innovative solutions to the actual technological demands. Further and deeper research is also needed to produce starters with novel properties and solve limitations such as metabolic activity and microbial stability, susceptibility to bacteriophage infections, spontaneous mutations or loss of key-physiological properties and sensorial acceptance by consumers. Particularly, the development of sourdough starter cultures for bread making is very topical due to the need to avoid deviations in bread quality between batches as frequently observed when the laborious and time-consuming artisanal procedures based on spontaneous sourdough fermentation are employed. In these artisanal processes, a piece of the spontaneously fermented dough, called mother-dough or sponge-dough, is kept aside and added to dough in next fermentation batch, thus serving as a natural ferment or microbial starter culture. In addition to the previous benefits, the use of starter cultures in bread making and other fermented food may be of interest to attain several other advantages, such as the reduction of production costs, fermentation times and risk of spoilage, to increase shelf-life, to predict microbial metabolic activities and to improve the control of the biotechnological processes, and to improve sensory quality and food safety, among others. Moreover, regarding the use of sourdough biotechnology, behind the technological advantages, it also holds a high potential to improve nutritional value and health-promoting effects of the final food products, including reduction of the glycemic response, increase of minerals bioavailability and promoting the formation of bioactive compounds (e.g., prebiotic oligosaccharides) [1,2,67].

Similarly, a considerable number of health benefits have been postulated as a result of the intake of viable LAB strains (probiotics) and which were correlated with their antimicrobial properties, including desirable modification of microbiota, prevention of pathogens, stimulation of immune system, immune modulation of the human host [68,69]. However, each property is strain-specific, as well as culture medium and/or environmental-dependent. The current study also showed that different experimental conditions (specifically, the antimicrobial evaluation of LAB by using the agar well diffusion method and liquid medium) can lead to different behaviors. However, it must be mentioned that trends on LAB inhibition properties remained similar in the both experimental conditions. Finally, it is apparent from this study that the isolation and characterization of LAB from different matrices and geographical locations are very likely to contribute to the discovery of a greater diversity of LAB with distinct phenotypic features.

### 3.4. Antifungal Activity of the Isolated Sourdough Lactic Acid Bacteria Strains

The antifungal activity of sourdough LAB against the species of *Aspergillus fischeri*, *Aspergillus nidulans*, *Penicillium oxalicum*, *Penicillium funiculosum*, *Fusarium poae*, *Alternaria alternate*, and *Fusarium graminearum* are displayed in Table 4. Delay of *Aspergillus fischeri* spore formation was observed by using the sourdough LAB isolates *L. farraginis* No. 206, *P. acidilactici* No. 29, and *L. paracasei* No. 244 strains. The most sensitive molds to LAB presence were *Aspergillus nidulans*, *Penicillium funiculosum* and *Fusarium poae* fungi strains. *Aspergillus nidulans* was suppressed by all sourdough-originating LAB strains. Nonetheless, the most effective inhibition of mycelium growth and sporulation with a large clear DIZ around the punched well was established for *L. curvatus* No. 51.

A very good inhibition of *Penicillium funiculosum* mycelium growth and sporulation was found with the inoculation of *L. plantarum* No. 122, *P. pentosaceus* No. 183, *P. acidilactici* No. 29, *L. paracasei* No. 244, *L. plantarum* No. 135, *L. coryniformins* No. 71, and *L. uvarum* No. 245. The strongest inhibition of *Fusarium poae* was obtained by the application of *L. plantarum* No. 122, *L. casei* No. 210, *L. farraginis* No. 206, *L. paracasei* No. 244, and *L. coryniformins* No. 71. The *Penicillium oxalicum* was inhibited by all the tested sourdough LAB strains but *P. acidilactici* No. 29 and *L. brevis* No. 173–and where the highest inhibition of mycelium growth and sporulation was obtained with *Lactobacillus plantarum* No. 122. Furthermore, the *Alternaria alternata* and *Fusarium graminearum* were suppressed by 7 and 5 sourdough LAB strains, respectively, with the highest inhibition of both fungi with *L. plantarum* No. 122. Delay of *Alternaria alternata* spore formation with a small clear DIZ around the punched well was unfolded by using *P. acidilactici* No. 29 and *L. coryniformins* No. 71.

Fungal spoilage of food and feed represents a major concern, as well as for human and animal health. Significant progress has been reported on the isolation and characterization of antifungal compounds (different organic acids, peptides, fatty acids, etc.), as well as various food-based applications of antifungal LAB have been described in the literature [21,23,26,27,70]. Rouse et al. [52] reported four cultures with antifungal activity originally isolated from cereals, chiefly strains of *L. plantarum* (CM8) and *P. pentosaceus* (R47) [71]. Other strains belonging to *L. plantarum* and *P. pentosaceus* species have previously been found to have antifungal activity [72]. Antifungal activity of the above-mentioned studies [71] was explained by different amounts of organic acids yielded by LAB strains and detected in the supernatants. Also, Magnusson et al. [72] unveiled the existence of cyclic dipeptides metabolized by LAB and with antifungal activity. According to Rouse et al. [71], by varying the growth conditions and chemical composition of fermentable media, the LAB antifungal activity is fairly consistent and stable [23]. The anti-fungal activity of these microorganisms is, obviously, modulated by such growth parameters as temperature, pH and incubation time. From above it is intuitive that LAB, which possess antifungal activity and are generally regarded as safe microbial starters, may represent an important tool to control or retard mold growth in a wide range of applications [1,2,3,4,10].

The production of organic acids during sourdough fermentation constitutes, indeed, a major safeguard against pathogens and spoilage microorganisms, since their undissociated forms exhibit strong microbial antagonistic effects, as verified in the present research effort. The organic acid production increases the mold-free shelf-life of sourdough breads. In LAB, the anti-microbial activities of lactic and acetic acids, at a given molar concentration, are not the same; the latter is more inhibitory than lactic acid, and can inhibit yeasts, molds and bacteria. Still, propionic acid inhibits preferentially fungi and bacteria, thus the co-fermentation of propionic acid bacteria, LAB and yeasts seems to be a promising natural sourdough-based biotechnological method to retard mold growth (although some technical limitations may occur) [1,2,3,4,10].

Compounds with anti-fungal properties are typically low-molecular-weight molecules, as is the case of organic acids, reuterin (β-hydroxypropionaldehyde), hydrogen peroxide, as well as proteinaceous compounds, hydroxyl fatty acids and phenolic compounds. Since fungistatic effects are mainly due to acetic rather than lactic acid production (acetic acid has a higher dissociation constant than lactic acid), heterofermentative LAB display the widest spectrum of anti-fungal activity, which is in agreement with the current experimental data [23,27]. Furthermore, the main LAB metabolites bearing anti-fungal attributes are, besides lactic and acetic acids, carbon dioxide, diacetyl, hydrogen peroxide, caproic acid, 3-hydroxy fatty acids, phenyllactic and 4-hydroxy-phenyllactic acids, cyclic dipeptides, fungicins (i.e., compounds of proteinaceous nature) and reuterin [1,2,3,4,10].

In Western Europe, economic losses related to contamination by molds in bread are estimated to exceed the 200 M€ a year. Mold growth may produce many kinds of food spoilage, viz. off-flavors, toxins, discoloration, rotting and triggering of pathogenic or allergenic effects. Though, production of mycotoxins is the most important issue of mold spoilage of foods. The experimental data in this work showed that sourdough biotechnology and sourdough LAB may play important roles on this purpose, and were the main LAB possessing ability to prevent (or limit) mycotoxinogenic mold growth belong to the genera of *Lactobacillus*, *Lactococcus*, *Pediococcus*, and *Leuconostoc* [4].

## 4. Conclusions

Spontaneous sourdough is an excellent source of lactic acid bacteria (LAB), as demonstrated in the present study, with high potential to answer to several needs and overcome technical limitations faced in the industry. The antimicrobial and antifungal properties as well as the metabolic capacity to ferment a large number of carbohydrate sources are only some good examples of the high techno-economic industrial potential of sourdough LAB or sourdough starter cultures based on single cultures or in distinct consortium designs.

In the present research work, 13 LAB strains with potential industrial application were isolated from spontaneous fermented rye sourdough. Most of the isolated sourdough strains showed versatile carbohydrate metabolisms. *Leu. mesenteroides* No. 242 and *L. brevis* No. 173 demonstrated ability for gas production. The seven out of 13 isolated strains exhibited growth yieldshigher than 7.0 log cycle after being submitted for 2 h to a culture medium with a pH value of 2.5. Furthemore, *L. plantarum* No. 122, *L. casei* No. 210, *Lactobacillus curvatus* No. 51, *L. paracasei* No. 244 and *L. coryniformins* No. 71 revealed inhibition properties against all the tested 15 pathogenic and opportunistic bacterial strains. Also, most of the isolated sourdough LAB displayed antifungal activities against seven selected mold strains. These results unveiled that LAB isolated from spontaneous sourdough are promising antimicrobial and antifungal ingredients.

This work highlighted the potential of sourdough biotechnology and, particularly, the LAB isolated from spontaneous sourdough to be applied in a wide range of agri-food industries, such as baking (sourdough bread and other sourdough-based baking goods, e.g., biscuits, cookies, crackers, pastry, pizza and pasta), feed and pet food, dairy (yoghurts, cheeses, smoothies, etc), meat (dried sausages, etc), alcoholic beverages (e.g., beer, cider and wine) and non-alcoholic beverages (juices, refrigerants, non-fermented and fermented cereal drinks), and nutraceuticals (dietary supplements and food additives), and also in other industrial sectors such as cosmetics and pharmaceuticals.

## Figures and Tables

**Table 1:** Primer details of PCR-based identification of LAB at genus and species level.

Genus and Species	References	Primers Used (Fw = Forward; Re = Reverse)	Size (pb)
***Lactobacillus***			
*Lactobacillus spp.*	[29]	Fw: 5′ CAA NTG GAT NGA ACC TGG CTT T3′	250
		Re: 5′ GCG TCA GGT TGG TGT TG3′	
*Lactobacillus plantarum*	[30]	Fw: 5′ GCT GGA TCA CCT CCT TTC 3′	248
		Re: 5′ ATG AGG TAT TCA ACT TAT G 3′	
*L* *actobacillus casei*	[31]	Fw: 5′ CAA NTG GAT NGA ACC TGG CTT T 3′	520, 350
		Re: 5′ GAC GGT TAA GAT TGG TGA C 3′	
*L* *actobacillus paracasei*		Fw: 5′ ACT GAA GGC GAC AAG GA 3′	520, 240
		Re: 5′ GCG TCA GGT TGG TGT TG 3′	
*L* *actobacillus curvatus*	[32]	Fw: 5′ GGA GGG TGT TCA GGA C 3′	260
		Re: 5′ GGA GGG TGT TGA TAG G 3′	
*L* *actobacillus brevis*	[33]	Fw: 5′ GCC TTG SGA GAT GGT CCT C 3′	502
		Re: 5′TTT GAC GAT CAC GAA GTG ACC G 3′	
***Leuconostoc***			
*Leuconostoc mesenteroides*	[34]	Fw: 5′ AAC TTA GTG TCG CAT GAC 3′	1150
		Re: 5′AGT CGA GTT ACA GAC TAC AA 3′	
***Pediococcus***			
*Pediococcus spp.*	[35]	Fw: 5′ GAA CTC GTG TAC GTT GAA AAG TGC TGA 3′	701
		Re: 5′GCG TCC CTC CAT TGT TCA AAC AAG 3′	
*Pediococcus pentosaceus*	[36]	Fw: 5′ CGA ACT TCC GTT AAT TGA TCA G3′	872
		Re: 5′ACC TTG CGG TCG TAC TCC 3′	
*Pediococcus acidilactici*		Fw: 5′ CGA ACT TCC GTT AAT TGA TTA T3′	449
		Re: 5′GTT CCG TCT TGC ATT TGA CC 3′	

**Table 2:** Bands of the isolated LAB genus (analyzed by the BioNumerics v4.0 software package).

**100bp DNA-Ladder Extended**	***Leuco-nostoc mesente-roides* No. 242**	***Lactoba-cillius corynifor-mins* No. 71**	***Lactoba-cillius curvatus* No. 51**	***Pediococcus pentosaceus* No. 183**	***Lactoba-cillius brevis* No. 173**	***Lactoba-cillius plantarum* No. 135**
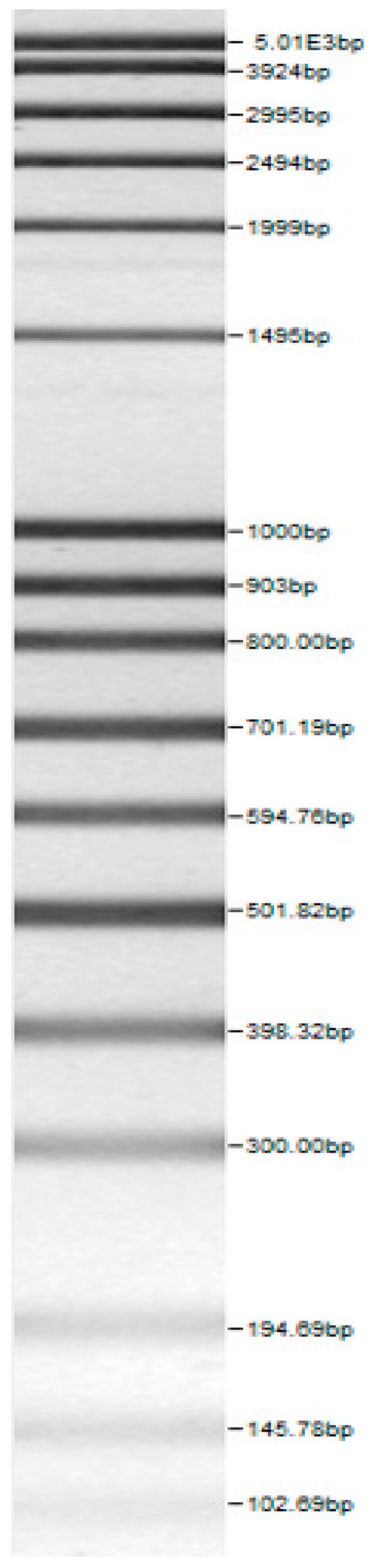	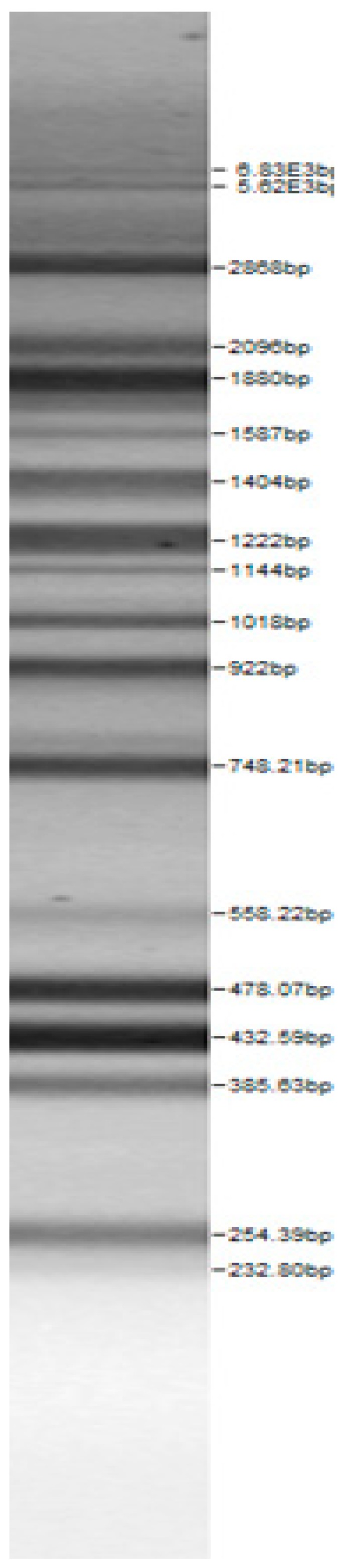	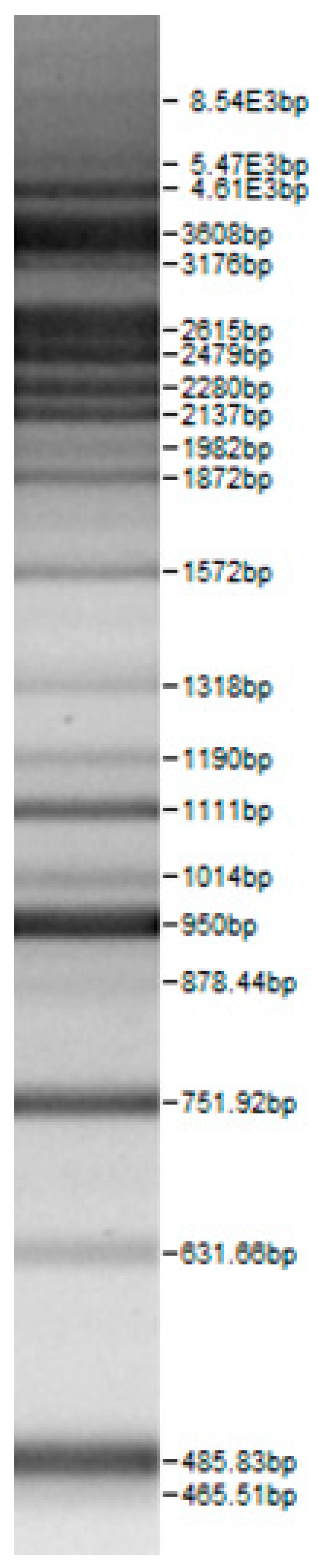	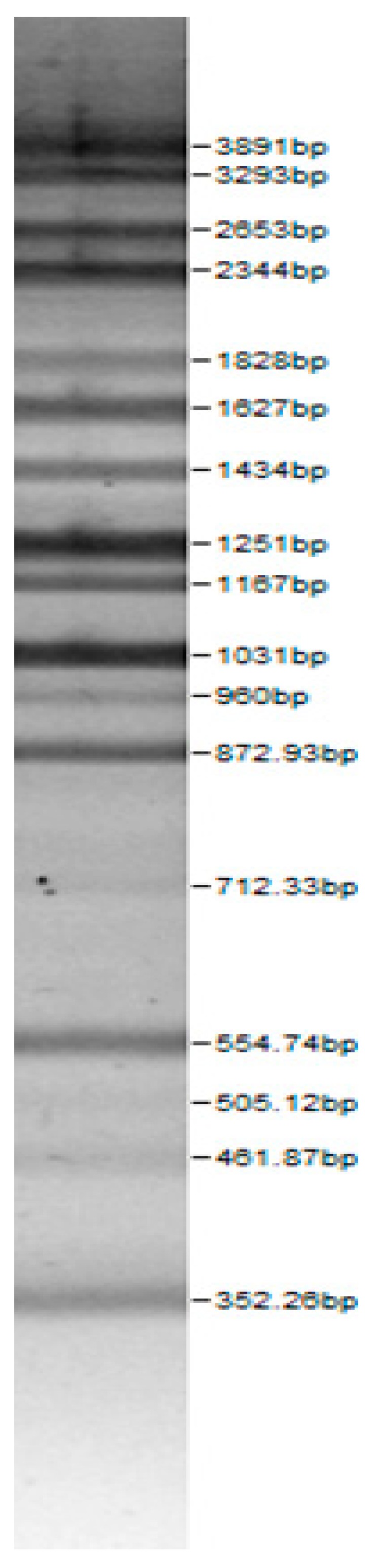	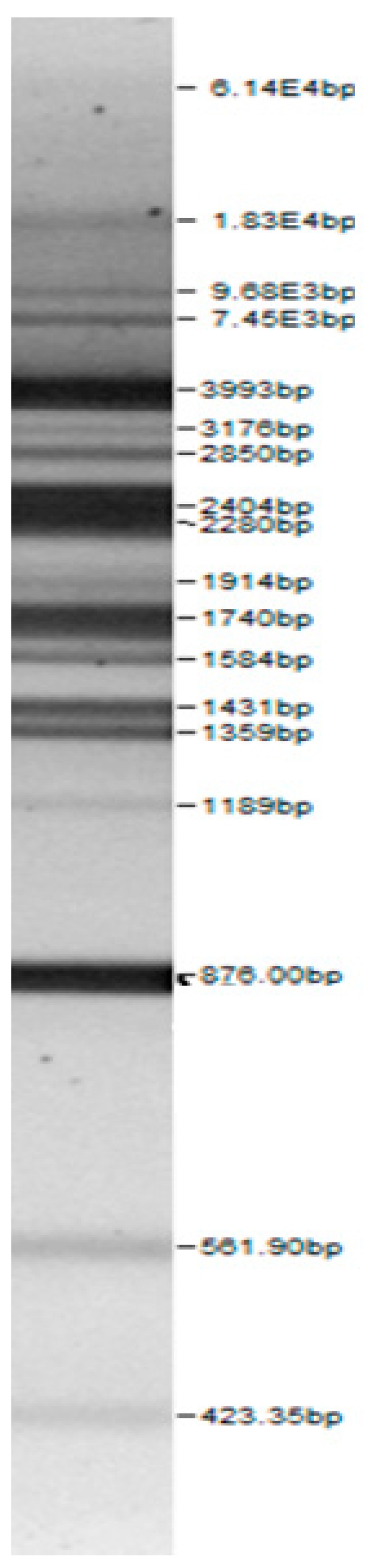	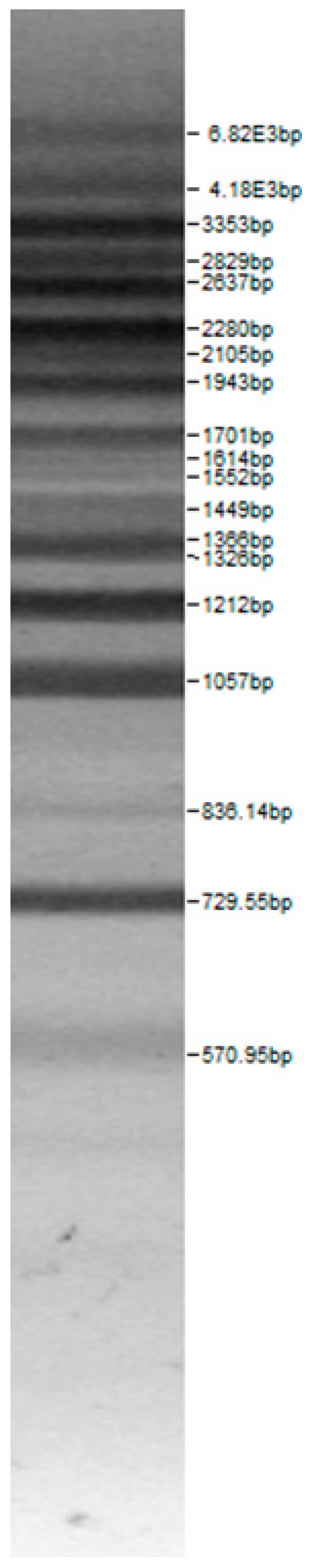	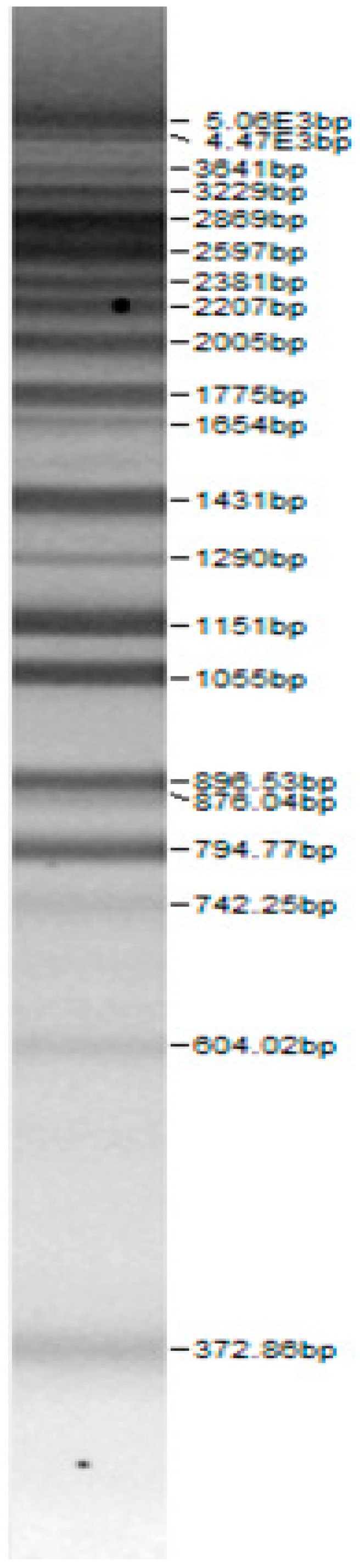
***Lactoba-cillius uvarum* No. 245**	***Lactoba-cillius plantarum* No. 122**	***Lactoba-cillius casei* No. 210**	***Leuconos-toc mesenteroi-des* No. 225**	***Lactobacillius farraginis* No. 206**	***Pedioco-ccus acidilac-tici* No. 29**	***Lactobaci-llius paracasei* No. 244**
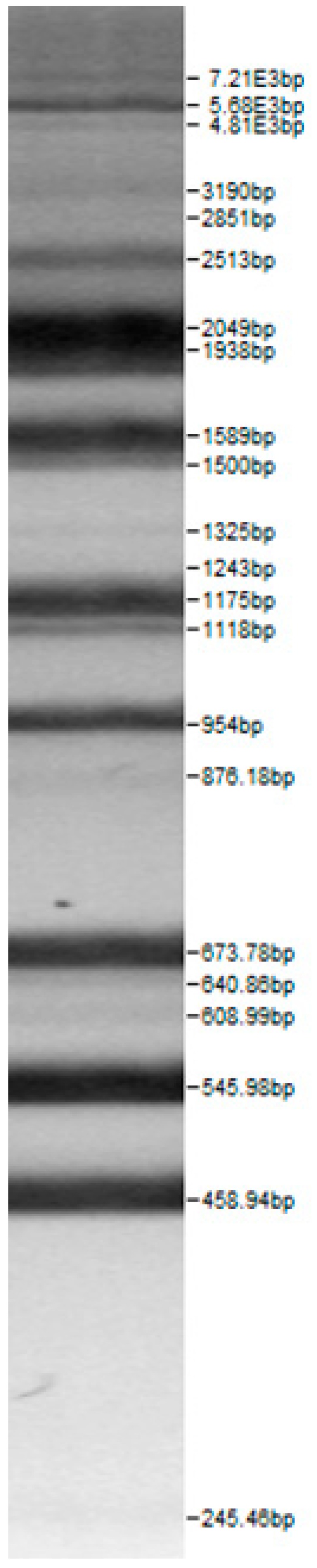	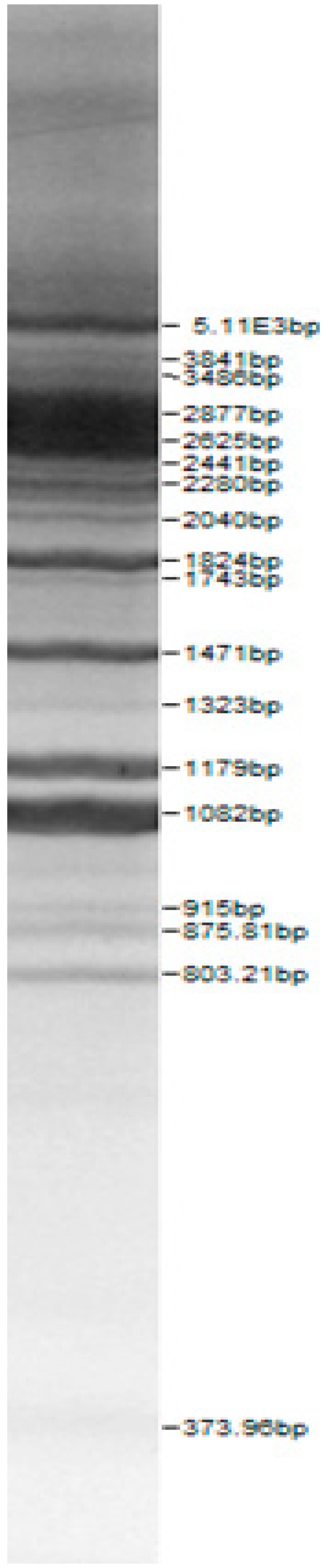	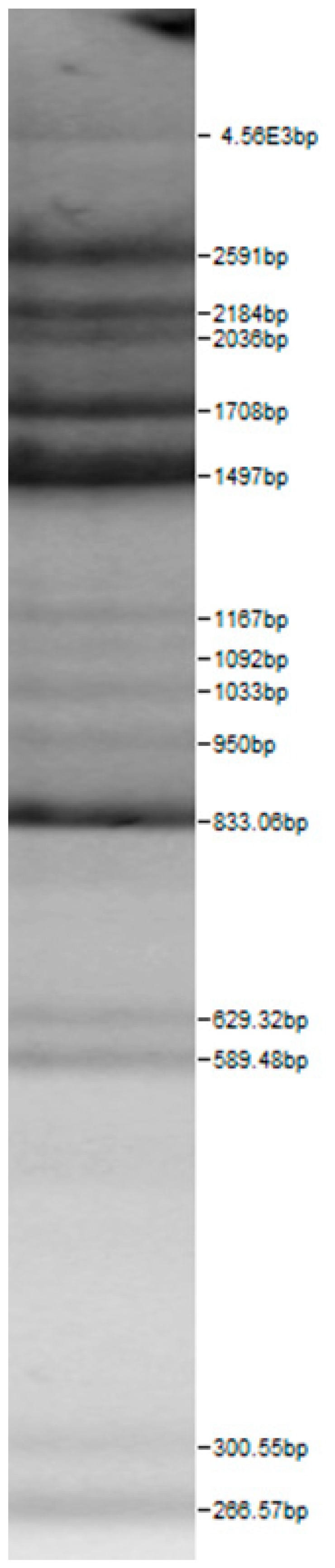	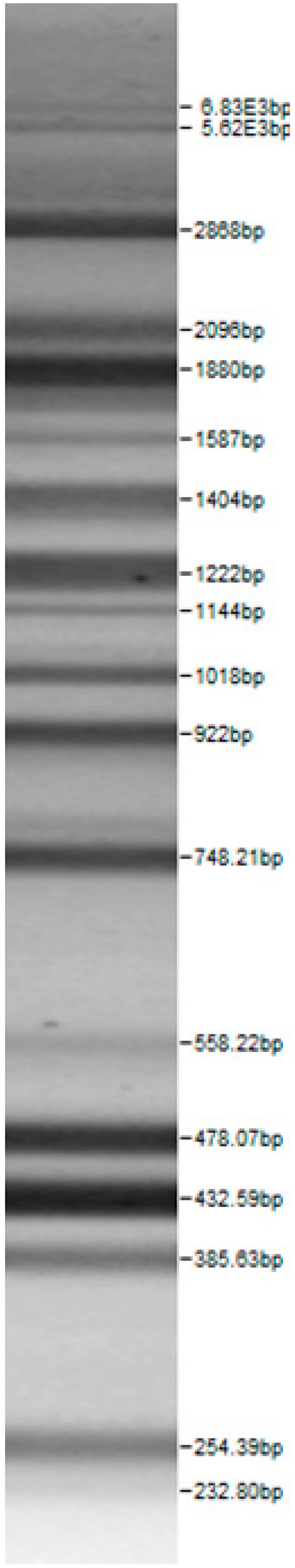	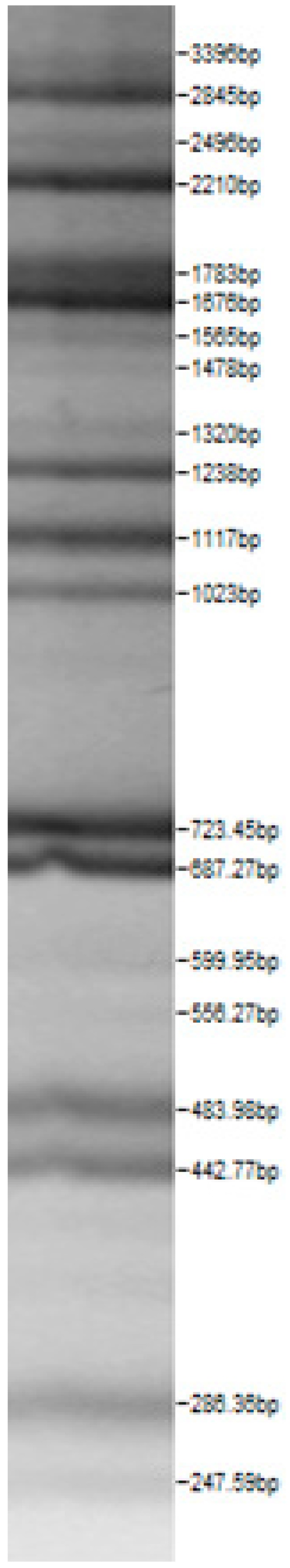	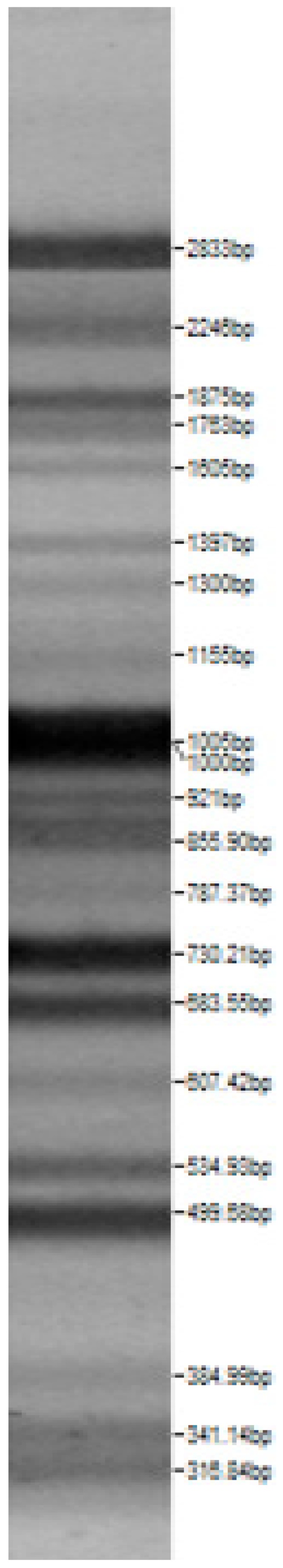	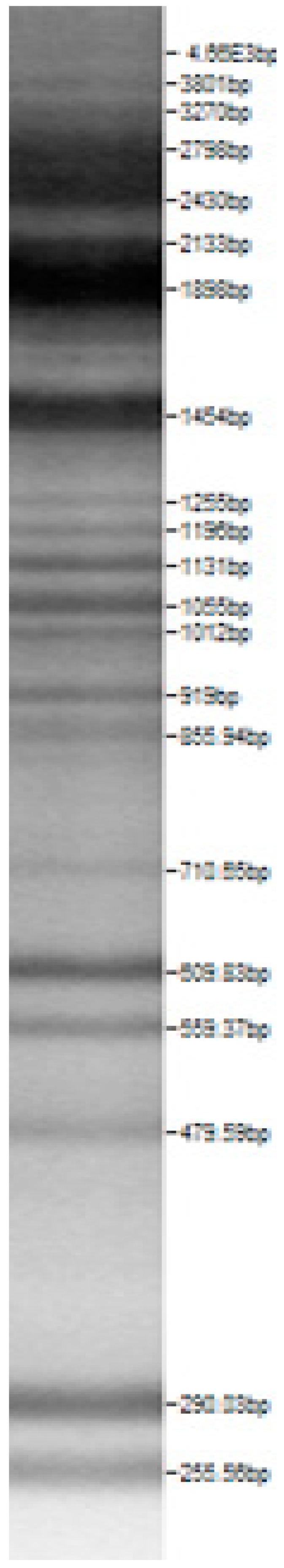

**Table 3:** Carbohydrate metabolism, gas production, tolerance to temperature (10, 30, 37 and 45 °C) and low pH conditions (pH 2.5 for 2 h) of the isolated sourdough lactic acid bacteria (LAB) strains.

	*Leu.mesenteroi-des* No. 242		*L. coryniformins* No. 71	*P. pentosaceus* No. 183	*L. plantarum* No. 122	*L. curvatus* No. 51	*L. casei* No. 210	*L. brevis* No. 173	*L. uvarum* No. 245	*Leu.mesenteroi-des* No. 225	*L. farraginis* No. 206	*L. plantarum* No. 135	*P. acidilactici* No. 29	*L. paracasei* No. 244
Glicerol	−		−	−	−	−	−	−	−	−	−	−	−	−
d-arabinose	−		−	−	−	−	−	−	−	−	−	−	−	−
l-arabinose	+++		+++	+++	+++	+++	−	+++	+++	−	+++	+++	+++	−
d-ribose	+++		+++	+++	+++	+++	+++	+++	+++	−	+++	+++	+++	+++
d-xylose	+++		−	−	−	−	−	+++	−	−	+++	−	+++	−
l-xylose	−		−	−	−	−	−	−	−	−	−	−	−	+++
d-adonitol	−		−	−	−	−	−	−	−	−	−	−	−	+
Methyl-ßd-xYlopiranoside	−		−	−	−	−	−	−	−	−	−	−	−	−
d-galactose	+++		+++	+++	+++	+++	+++	+++	+++	−	+	+++	+++	+++
d-glucose	+++		+++	+++	+++	+++	+++	−	+++	+++	−	+++	+++	+++
d-fructose	+++		+++	+++	+++	+++	+++	+++	+++	+++	+++	+++	+++	+++
d-mannose	+++		+++	+++	+++	+++	+++	−	+++	+++	−	+++	+++	+++
l-sorbose	−		−	−	−	−	−	−	−	+	−	−	−	−
l-rhamnose	−		+	++	+	−	−	−	−	−	−	+	++	+++
Dulcitol	−		−	−	−	−	+++	−	−	−	−	−	−	+++
Inositol	−		−	−	−	−	−	−	−	−	−	−	−	−
d-mannitol	+		+++	−	+++	+++	+++	−	+++	+++	−	+++	−	+++
d-sorbitol	−		+++	−	+++	+++	+++	−	+++	−	−	+++	−	+++
Methyl-αD-mannopyranoside	−		++	−	+++	+	−	−	+	−	−	+++	−	−
Methyl-αD-glucopyranoside	+++		−	−	−	−	+++	−	−	+++	−	+	−	+++
*N*-acetylglucosamine	+++		+++	+++	+++	+++	+++	−	+++	+++	−	+++	+++	+++
Amigdalin	+++		+++	+++	+++	+++	+++	−	+++	+++	−	+++	+++	+++
Arbutin	−		+++	+++	+++	+++	+++	−	+++	+++	−	+++	+++	+++
Esculin	+++		+++	+++	+++	+++	+++	−	+++	+++	−	+++	+++	+++
Salicin	+++		+++	+++	+++	+++	+++	−	+++	+++	−	+++	+++	+++
d-cellobiose	+++		+++	+++	+++	+++	+++	−	+++	+++	−	+++	+++	+++
d-maltose	+++		+++	+++	+++	+++	++	+++	+++	+++	+++	+++	−	+++
d-lactose	−		+++	−	+++	+++	−	−	+++	−	−	+++	+++	+++
d-melibiose	+++		++	+++	+++	−	−	−	−	−	+++	+++	−	−
d-saccharose	+++		+++	+++	+++	+++	+++	−	+++	+++	−	+++	+++	+++
d-trehalose	+++		+++	+++	+++	+++	+++	−	+++	+++	−	+++	+++	+++
Inulin	−		−	−	−	−	++	−	−	−	−	−	−	+++
d-melezitose	−		+++	−	+++	+++	+++	−	+++	−	+++	+++	−	+++
d-raffinose	+++		−	+++	+++	−	−	−	−	−	−	−	−	−
Amidon	−		−	−	−	−	−	−	−	−	−	−	−	−
Glycogen	−		−	−	−	−	−	−	−	−	−	−	−	−
Xylitol	−		−	−	−	−	−	−	−	−	−	−	−	−
Gentiobiose	++		+++	+++	++	++	++	−	++	+++	−	++	+++	+++
d-turanose	−		−	−	+++	+++	+++	−	+++	+++	−	+++	−	+++
d-tagatose	−		−	+++	+++	−	+++	−	−	−	−	+++	+++	+++
d-fucose	−		−	−	−	−	−	−	−	−	−	−	−	−
l-fucose	−		−	−	−	−	−	−	−	−	−	−	−	−
d-arabitol	−		−	−	−	−	−	−	−	−	−	−	−	−
l-arabitol	−		−	−	−	−	−	−	−	−	−	−	−	−
Potassium gluconate	+		−	−	+	+	+	+	+	−	++	++	+	++
Potassium 2-ketogluconate	−		−	+	−	−	−	−	−	−	−	−	−	−
Potassium 5-ketogluconate	−		−	−	−	−	−	++	−	−	++	−	−	−
Gas production (+/−)	+		−	−	−	−	−	+	−	−	−	−	−	−
Temperature tolerance	10 °C	−	−	−	−	−	+	−	−	−	−	−	−	−
	30 °C	+	+++	++	++	+	+++	+	++	++	+	++	++	++
	37 °C	−	+	++	+	+	+++	−	++	++	+	+	+	++
	45 °C	−	−	−	+	−	+	−	−	−	++	+	+	−
pH 2.5	0 h log (CFU/mL)	8.14 ± 0.2 ^c^	6.51 ± 0.3 ^a^	7.97 ± 0.1 ^c^	8.43 ± 0.3 ^d^	8.31 ± 0.2 ^c,d^	8.47 ± 0.3 ^d^	8.86 ± 0.2 ^e^	9.03 ± 0.2 ^e^	8.14 ± 0.1^c^	8.51 ± 0.2 ^d^	8.08 ± 0.2 ^c^	7.5 ± 0.2 ^b^	9.41 ± 0.2 ^f^
	2 h log (CFU/mL)	2.69 ± 0.1 ^a^	n.d.	7.40 ± 0.1 ^d^	5.72 ± 0.2 ^c^	3.5 ± 0.1 ^b^	8.36 ± 0.2 ^e^	8.67 ± 0.1 ^e^	7.55 ± 0.2 ^d^	2.69 ± 0.2 ^a^	8.42 ± 0.1^e^	7.69 ± 0.1 ^d^	3.2 ± 0.1 ^b^	9.29 ± 0.1 ^f^

Interpretation of lactic acid bacteria (LAB) growth in API 50 CH system and API 20 E system: +++ = strong growth (yellow); ++ = moderate growth (green); + = weak growth (dark green); − = no growth (blue); n.d. = not determined. ^a–f^ Mean values with different letters are significantly different (*p* ≤ 0.05).

**Table 4:** Inhibition of mold strains by the isolated sourdough lactic acid bacteria (LAB) strains.

Isolated Sourdough Lactic Acid Bacteria (LAB) Strains	*Aspergillus fischeri*	*Aspergillus nidulans*	*Penicillium oxalicum*	*Penicillium funiculosum*	*Fusarium poae*	*Alternaria alternata*	*Fusarium graminearum*
*Leuconostoc mesenteroides* No. 225	−	++	+	+	++	−	−
*Lactobacillus plantarum* No. 122	−	++	+++	+++	+++	++	++
*Enteroccocus pseudoavium* No. 242	−	+	+	+	++	−	−
*Lactobacillus casei* No. 210	−	++	+	++	+++	+	+
*Lactobacillus curvatus* No. 51	−	+++	++	++	++	−	−
*Lactobacillus farraginis* No. 206	+	+	++	+	+++	+	−
*Pediococcus pentosaceus* No. 183	−	++	++	+++	+	+	−
*Pediococcus acidilactici* No. 29	+	++	−	+++	++	++	−
*Lactobacillus paracasei* No. 244	+	+	+	+++	+++	−	+
*Lactobacillus plantarum* No. 135	−	++	+	+++	++	+	+
*Lactobacillus coryniformins* No. 71	−	++	++	+++	+++	++	−
*Lactobacillus brevis* No. 173	−	+	−	++	++	−	−
*Lactobacillus uvarum* No. 245	−	++	+	+++	+	−	+

Legend: (−) No inhibition; (+) Delay of spore formation; (++) Delay of spore formation with a small clear zone of inhibition around the punched well; (+++) A very good inhibition of mycelium growth and sporulation with large clear zones around the punched well.
